# Book Reviews

**Published:** 1831-12

**Authors:** 


### CRITICAL ANALYSES.

Quæ laudanda forent, et quæ culpanda, vicissim

Illa, prius, cretâ; mox hæc, carbone, *notamus*.—PERSIUS.

*A Manual of Medical Jurisprudence, compiled from the best Medical and Legal Works: comprising an Account of* 1, *the Ethics of the Medical Profession;* 2, *the Charters and Statutes relating to the Faculty; and,* 3, *all Medico-legal Questions, with the latest Decisions: being an Analysis of a Course of Lectures on Forensic Medicine, annually delivered in London, and intended as a Compendium for the use of Barristers, Solicitors, Magistrates, Coroners, and Medical Practitioners.* By MICHAEL RYAN, M.D., Member of the Royal College of Physicians in London, &c.—8vo. pp. 327. Renshaw and Rush, London.

WE have received much less, both of pleasure and profit, than we anticipated, in the perusal of this volume: it dips into and touches so many points, without minutely considering or clearly explaining any, that it reminds us of the wayward wanderings of an unfortunate bee, who, compelled by natural instinct to sip nectar from flowers of the same kind alone during each excursion, lights early in the morning upon some solitary plant, whose fellow it meets not with during the remainder of the day, and thus, although it visit many, and appear most busy to all beholders, returns to the hive at night with much fatigue, but with little honey. We shall, therefore, not pretend to analyse the work before us, because we think the manipulation would be almost as tedious as in a case of suspected poisoning, where some small quantity of an unknown active ingredient is mixed with, and all but lost, amongst a mass of crude ingesta; and as there is no bill of indictment pending against the author, we shall content ourselves with picking out here and there such particles as soonest catch the eye.

We wonder whose lectures are alluded to in the following sweeping paragraph: we know but of one course delivered in London, of which the author has had sufficient opportunity of judging before he condemns; for several we can vouch that the condemnation is unjust.

"It is perfectly impossible to comprehend the policy of those who regulate medical education in this section of the nation, and exclude the study of the subjects under consideration. It is right, however, to disabuse the minds of students in a very erroneous idea which they entertain, and which is indeed unfairly impressed upon them by teachers, namely, that when they have obtained the testimonials of the respective medical authorities, fame and fortune are easily attained, as the public will appreciate the dignity, rights, and privileges conferred by the corporations. This is a gross delusion, and contrary to the opinions of the most eminent and honourable members of the profession, who have ever unanimously declared, that it is one thing to be fascinated with the study of the sublime and interesting science of giving health to man, but another to engage in its practice, successfully to overcome the objections made to youth, and the innumerable difficulties which impede the young practitioner. These obstacles are only to be removed by a profound knowledge of science, which leads to a perfect knowledge of mankind, and by the due observance of those moral duties which inculcate the highest principles of virtue and honour, and have been always universally allowed to distinguish men profoundly acquainted with the noble science of medicine. It will appear in the course of these observations, that a perfect knowledge of public or forensic medicine is indispensable to medical practitioners. If these conclusions be admitted, is it not a matter of astonishment that medical students in every part of this empire never hear a single observation during their education on the ethical duties they owe the profession and the public. A man who obtains the degree of M.D., the diploma for surgery, or the licence for pharmacy, is as ignorant of medical ethics as he is of the cause of the perpetual motion; he is ushered into practice without the slightest acquaintance with the moral and delicate duties which he has to perform in the different situations in society in which he will be placed. Hence the dishonourable conduct for which the profession in this age is so remarkably distinguished, and hence the chief cause of the humiliation and degradation of the noblest of the human sciences in the estimation of the public." (P. 2.)

Upon this point we would, if it were needed, immediately join issue with our author, and we boldly deny the statement that the "teachers," with whom we have come in contact, ever "unfairly impressed," or indeed impressed at all, upon the minds of students the very erroneous idea, (which we likewise doubt whether students in general entertain,) "that when they have obtained the testimonials of the respective medical authorities, fame and fortune are *easily* obtained;" and the only "gross deception" that we perceive in the question is that which is here created, that the author may shew his Quixotism in its destruction. Again, from what parabolic mirror can the passing events of the present day be reflected to the writer's mind, that he should think himself justified to assume "the humiliation and degradation of the noblest of the human sciences in the estimation of the public," and to talk of "the dishonourable conduct for which the profession in this age is so remarkably distinguished."

The propositions are so absurd, that their mere statement to all who live in the world, and know any thing of the world, will at once be a full refutation; and without fear of contradiction we assert, that the last quarter of a century has wrought greater changes and improvements in the medical profession, and advanced medicine and medical practitioners more in the estimation of the public, than any consecutive five and twenty years that the author, with all his love for antiquity, can point out. The medical profession humiliated and degraded, indeed, when it has been improving and advancing with an almost incredible rapidity! for as to the few apostates from honour and from truth, who, as soon as they have appeared, have been vigorously cast off like sloughs from a healthy sore, they deserve not to be had in remembrance, further than as shades, which shew the stronger from their contrast with the general clear and honourable conduct to which they are the background, and without which they would be as much disregarded as, now they are known, they are deservedly despised.

As a specimen of the simplicity and purity of the author's style, we select the following:

"It is an extraordinary fact, that in the united kingdom, where the various branches of medical science are so highly cultivated, that the study of state medicine should be neglected and disregarded; though it ranges over the vast field of medical erudition, extending through the several regions of the universe, the animal, vegetable, gaseous, and mineral kingdoms; in a word, from the azure fluid above to crude matter below, and comprehending the whole of the fascinating sciences included in medicine: yet there is no situation in which a medical practitioner can be placed, that requires a more cautious and judicious discharge of his duties than in judicial proceedings: the whole of his elementary studies are called into operation, general anatomy, physiology, semeiology, pathology, and chemistry." (P. 3.)

We should like to see the authorities on which the assertion contained in the first sentence of the first chapter is founded: "In the beginning of the world, man was endowed with a knowledge of the innoxious and noxious properties of all the productions of the creation."

We are no phrenologists, yet, as honest men, we must enter our protest against the following tirade, which is as destitute of truth as it is inconclusive in its deductions.

"The doctrine of the materiality and mortality of the soul, which is that of materialism and phrenology, should for ever be exploded as totally false, and unworthy of all regard, as subversive of the fundamental principles of all religions, as introducing civil anarchy into the political economy of legislation, as substituting disorder for harmony, despair for hope, and eternal darkness for everlasting light.

"If materialism tended to promote the happiness of society, to assist our hopes, to subdue our passions, to instruct man in the happy science of purifying the polluted recesses of a vitiated heart, to confirm him in his exalted notion of the dignity of his nature, and thereby to inspire him with sentiments averse to whatever may debase the excellence of his origin, the public and the medical profession would be deeply indebted to the phrenologists. But the tendency of phrenology, however disguised, is to make mind a secretion or function of the brain, and thus to deny the immortality of the soul. Unbelievers, in general, wish to conceal their sentiments; they have a decent respect for public opinion, are cautious of affronting the religion of their country, fearful of undermining the foundations of civil society. Some few have been more daring, but less judicious, and have, without disguise, professed their unbelief, and again retracted their opinions. In denying the immortality of the soul, they deny the authenticity of the Bible; they sap the foundations of all religions; they cut off at one blow the merit of our faith, the comfort of our hope, and the motives of our charity. In denying the immortality of the soul, they degrade human nature, and confound man with the vile and perishable insect, and overturn the whole system of religion, whether natural or revealed. In denying religion, they deprive the poor of the only comfort which supports them under their distresses and afflictions, and wrest from the hands of the powerful and rich the only bridle to their injustice and passions; and pluck from the hearts of the guilty the greatest check to their crimes, that remorse of conscience which can never be the result of a handful of organized matter, that interior monitor, which makes us blush in the morning at the disorders of the foregoing night, and which erects in the breast of the tyrant a tribunal superior to his power. Such are the consequences naturally resulting from the principles laid down in phrenological writings. It is no intention of ours to fasten the odium of infidelity on any portion of our profession; but it surprises us that men, whose understandings have been enlightened by the Christian revelation, and enlarged by the study of medicine (the most extensive and varied of all human sciences,) should broach tenets which equally militate against the first principles of reason and the oracles of the Divinity; and which, if true, would be of no service to mankind. Of what benefit to humanity would be the establishment of phrenology? We answer none; but, on the contrary, the greatest injury. If any man be so unhappy as to work himself into the conviction that his soul is a function or a secretion of the brain, and of course must perish with it, he would still do well to conceal this horrid belief with more secrecy than the druids concealed their mysteries. In doing otherwise, he only brings disgrace upon himself; for the notion of religion is so deeply impressed on our minds, that the bold champions who would fain destroy it are considered by the generality of mankind, and our profession, as public pests, spreading disorder and mortality wherever they appear; and in our feelings we discover the delusions of a cheating and unmedical philosophy, which can never introduce a religion more pure than that of the Christian's, nor confer a more glorious privilege on man than that of an immortal soul. In a word, if it be a crime to entertain such a doctrine, it is consummate folly to boast of it. Whence this eagerness to propagate systems, the tendency whereof is to slacken the reins that curb the irregularity of our desires, and restrain the impetuosity of our passions? It must proceed from a corruption of the human heart, averse to restraint, or from the vanity of the mind, which glories in striking from the common path, and not thinking with the multitude. In vain are the phrenologists informed by the anatomist, that he can find bile in the liver, urine in the kidneys, but none of the faculties of the mind in the brain. In vain are they told that after death, when volition has ceased, the motions of the muscles can be excited by galvanism, and that, though muscular motion be restored, we cannot recal volition, or the other mental faculties: rather a strong proof that motion and volition are not exactly the same thing. These and ten thousand other proofs are lost on the phrenologists: they set up the proud idols of their own fancies in opposition to the oracles of the Divinity; and in endeavouring to display absurdities in the Christian religion, fall into much greater. To them we can, with due deference, and without disclaiming our title to good manners, apply the words of St. Paul to the philosophers of his time: 'They became vain in their imaginations; professing themselves wise, they became fools.'" (P. 16.)

We profess not to advocate phrenology: as far as we have examined it, we believe it to be untenable; we think many of its conclusions drawn from too partial a summary of facts; that, if it does contain some truth, it does not contain the whole truth, and nothing but the truth. Still, though adverse to this doctrine, we should scorn to attribute to its disciples opinions which they deny, and principles which they disavow.

The extracts from the chapter on *Professional Conduct*, relative to hospital and other medical charities, and in *private practice*, we give as examples of the so-called medical ethics.

"The next point for consideration is this, ought a physician to consult with an apothecary or not? The Royal College of Physicians in London decide in the negative, as also the Dublin College. Dr. Grattan of Dublin, observes,

"'If physicians will consult with apothecaries, and meet them at the appointed hour, on successive days, during the whole course of a long protracted fever, what are the public to infer? The natural inference is, that the physician must derive some information from the apothecary, and that he does not consider the absence of the apothecary from his shop as a matter of any consequence. This again leads to other conclusions, until at last it is supposed that the apothecary, having seen so much of the physician's practice, must be as well qualified to prescribe as the physician himself. Of course, on all future occasions he is applied to, and the physician no more thought of, until symptoms of the most urgent nature have presented themselves, and the apothecary begins to consider it not altogether prudent in him to allow his patient to die, unattended by a physician.

"'After a physician has been thus called in over an irregular practitioner, and when he performs merely the part of a useless pageant in the gloomy scene which is soon to follow, his want of firmness, and of steady adherence to that candour which his duty to the profession and the public requires, by no means tends to promote even his private interests. He gives occasion to the very person, who perhaps objected to him in the first instance, to observe that there was little use in employing him, and that it was evident he could have done nothing more than had been done before he was consulted. Thus are the public deceived; thus is the respectability of medicine injured, and thus are more lives annually sacrificed than it would be possible to calculate.

"'The presence of an apothecary at a consultation can be of no use whatever to the patient, and is very often injurious. Physicians, in his presence, cannot deliberate as freely as they would do were they by themselves: they feel that they are under the surveillance of a person who may have a partiality towards one physician, and a prejudice against another, and who may pass what comment he pleases on their opinions and practice. The effect of this is, to create a degree of caution and reserve on their part, altogether inconsistent with the object of a consultation, and which often renders it little else than a mere matter of form.

"'The presence of the apothecary has also a decided influence over the physician with respect to the medicines which he prescribes, so that, however honest his intentions, he cannot avoid ordering more than he otherwise would. If a physician were to pay two successive visits to a patient, when an apothecary was in attendance, without prescribing any medicine, what would be the consequence? It would probably be suggested that he knew nothing of the disorder, or that he wished to protract its duration, by not ordering such medicines as some other physician had prescribed, with the greatest success, in a case exactly similar; that it was a proof of the greatest avarice and illiberality to take his fee for doing nothing; in short, that he ought to be immediately dismissed, and that Doctor —— should be sent for.'" (P. 68.)

"The profession, though brought to a degree of perfection hitherto unequalled, has its dignity and degrees so despicably fallen, that the most illiterate assume and usurp its titles, and the university graduate is almost ashamed to be styled doctor, since he must share his title in common with the surgeon, the apothecary, the chemist, the druggist, and the nefarious quack. Every man may style himself doctor, and impose on the public with impunity. Such is the state of physic in London, in 1830. The English apothecary, however, is as much 'sinned against as sinning.' He is obliged to receive a medical and surgical education, expend five years in acquiring pharmaceutical knowledge, and undergo examination, before he is legally qualified. He then commences his profession, and has the mortification to discover that any man may usurp his rights, by placing the words chemist and druggist over his door; he also learns that his illiterate rival, who has received no medical education, robs him of his real vocation, the composition of medicine, vends drugs at half the price he charges, compounds nearly all physicians' prescriptions, prescribes for the sick; in a word, is physician, surgeon, apothecary, and obstetrician. The Apothecaries' Company have the power to prevent all this abuse, if they would only do their duty. In Scotland, the surgeon-apothecary must be a licentiate of the Royal College of Surgeons of Edinburgh, and must have received an excellent medical and surgical education.

"In Ireland, the apothecary is not obliged to receive a medical or surgical education, though he practises every branch of the healing art, and has his peculiar rights infringed on, especially in the remote parts of the country, by his old colleague the grocer.

"Under all these circumstances, can it be expected that the regular physician or surgeon ought to meet the general practitioners of this empire, and those who assume the title of such, in consultation? The Colleges of Physicians and Surgeons have invariably decided in the negative. If the members of each branch of medicine received the same education, of course there could be no objection to their meeting in consultation; but this has never been the case, and therefore the law and the public have wisely decreed a distinction of medical practitioners, which no class of the faculty can destroy: that it is quite preposterous to attempt it, the recent history of medicine in this country amply testifies." (P. 70.)

More illiberal sentiments, whether conceived or adopted, we have never met with: we trust, notwithstanding assertions to the contrary, that they are peculiar, or, at any rate, that they are *presque unique*. How well do they contrast with the honourable feelings and liberal expressions of GREGORY and PERCIVAL, quoted by Dr. Ryan.

The first "defended the necessity of medical men being versed in all the branches of the healing art, and concludes by observing, 'Every department of the profession is respectable, when exercised with capacity and integrity. I only contend for an evident truth, either that the different branches should be separately professed, or, if one person will profess all, that he should be regularly educated to, and thoroughly master of all. I am not here adjusting points of precedence, or insinuating the deference due to degrees in medicine: as a doctor's degree can never confer sense, the title alone can never command regard; neither should the want of it deprive any man of the esteem and deference due to real merit. If a surgeon or apothecary has had the education, and acquired the knowledge of a physician, he is a physician to all intents and purposes, whether he has a degree or not, and ought to be respected and treated accordingly. In Great Britain, surgery is a liberal profession; in many parts of it, surgeons or apothecaries are the physicians in ordinary to most families, for which trust they are often well qualified by their education and knowledge, and a physician is only called where a case is difficult, or attended with danger. There are certain limits, however, between the two professions, which ought to be attended to, as they are established by the customs of the country, and by the rules of their several societies: but a physician, of a candid and liberal spirit, will never take advantage of what a nominal distinction and certain privileges give him over other men, who in point of real merit are his equals; and will feel no superiority but what arises from superior learning, superior abilities, and more liberal manners; he will despise those distinctions founded in vanity, self-interest, or caprice, and will be careful that the interests of science and of mankind shall never be hurt, on his part, by a punctilious adherence to formalities.'" (P. 49.)

The latter writes, " In the present state of physic in this country, where the profession is properly divided into three distinct branches, a connexion peculiarly intimate subsists between the physician and apothecary, and various obligations result from it: on the knowledge, skill, and fidelity, of the apothecary, depend, in a very considerable degree, the reputation, the success, and usefulness, of the physician. As these qualities, therefore, justly claim his attention and encouragement, the possessor of them merits his respect and patronage.

"The apothecary is, in almost every instance, the precursor of the physician; and, being acquainted with the rise and progress of the disease, with the hereditary constitution, habits, and disposition, of the patient, he may furnish very important information. It is in general, therefore, expedient, (and, when health or life are at stake, expediency becomes a moral duty,) to confer with the apothecary before any decisive plan of treatment is adopted; to hear his account of the malady, of the remedies which have been administered, of the effects produced by them, and of his whole experience concerning the juvantia and lædentia in the case. Nor should the future attendance of the apothecary be superseded by the physician; for if he be a man of honour, judgment, and propriety of behaviour, he will be a valuable auxiliary through the whole course of the disorder, by his attention to varying symptoms, by the enforcement of medical directions, by obviating misapprehensions in the patient or his family, by strenghtening the authority of the physician, and by being at all times an easy and friendly medium of communication. To subserve these important purposes, the physician should occasionally make his visits in conjunction with the apothecary, because he will have no intelligence to offer which does not fall under the observation of the physician himself; nor any opportunity of executing his peculiar trust, without becoming burthensome to the patient by multiplied calls and unseasonable assiduity.

"This amicable intercourse and co-operation of the physician and apothecary, if conducted with the decorum and attention to etiquette, which should always be steadily observed by professional men, will add to the authority of the one, to the respectability of the other, and to the usefulness of both. The patient will find himself the object of watchful and unremitting care, and will experience that he is connected with his physician, not only personally, but by a sedulous representative and coadjutor: the apothecary will regard the free communication of the physician as a privilege and mean of improvement; he will have a deeper interest in the success of the curative plans pursued, and his reputation will be directly involved in the purity and excellence of the medicines dispensed, and in the skill and care with which they are compounded." (P. 85.)

We are glad to be able, in one point, to fully agree with Dr. Ryan, that "it is quite contrary to the objects for which hospitals and dispensaries are founded, to render them subservient to those in affluent circumstances; an abuse which exists in every one of them. This is an imposition on charity, and a direct injury to the profession, yet the medical officers connive at it. It is a fact, which cannot be controverted, that a large proportion of the patients admitted into the hospitals, especially of this city, and relieved at dispensaries, are not real objects of charity, and are often the relatives or personal friends of the governors or subscribers; and thus the junior members of the profession are seriously injured. This abuse exists in every part of the empire, but to a vast extent in this metropolis." (P. 61.)

The evil here complained of we have ourselves often noticed as a most unpardonable error on the part of those governors who constitute Boards for the regulation of the expenditure of funds, devoted (or intended to be devoted) to charitable purposes. We have frequently noticed the servants of noblemen and gentlemen possessing large fortunes, and whose domestics, if they fall sick in their service, may not unreasonably expect (for such has long been the custom in families of rank and fortune,) to have medical attendance provided for them: but now, instead of having it at home, they are despatched to the hospital or dispensary. This is not a mere casual occurrence, but of daily practice, and year after year it has been increasing; and we would venture to say that, at the moment we are writing, a large comparative number of the male in-patients, in every one of the London hospitals, would be found, on examination, to be noblemen and gentlemen's livery servants; for they are seldom refused admission, on their master's letters of recommendation being shewn, for fear that a refusal would stop their master's subscription, whose three or five guineas per annum, instead of being a charitable donation, is, in fact, a kind of health insurance for servants and dependents, which very frequently draws treble its amount from funds that the truly charitable have left for the benefit only of those who truly are in want. Thus, if a gentlemen sends his footman, or valet, or groom, into the hospital with acute rheumatism, probably brought on by staying out late at night, sitting or riding in wet weather, &c., the board, lodging, medicine, &c. of such a patient cannot be estimated at much less than a guinea per week, and if he remain in the hospital only six weeks, there is at once double the amount of the subscription taken from the funds of the hospital, not for the benefit of the poor, but to be put into the pockets of the rich: but this is not all; each small subscription entitles each subscriber to two or three presentations every year, so that the sum may probably be doubled, or even trebled, and a five-guinea subscription entitles a governor to have one patient always in the house, the burthen of whom upon the funds of the charity, if not truly an object for such relief, inevitably takes it from those who want it, and for whom it was designed, but who, friendless and powerless, have no other place of refuge, and thus are cruelly excluded from houses which are undoubtedly their own. Were a practice like this to be suddenly introduced, all would perceive its enormity; just as when, a short time since, it was endeavoured to quarter the sick police on the hospitals and dispensaries, by means of a few paltry subscriptions from the commissioners, the plan devised was at once seen through, and the proposition as universally rejected as it was made: still it differed little, and was perhaps less exceptionable than that which we now condemn, only the injustice was attempted to be done at once on the one hand, and the evil has been gradually creeping in on the other.

The notices of *Utero-gestation, Abortion, simulated, dissimulated, and imputed, Diseases*, &c. are all of them far too superficial: there are, however, many interesting cases detailed, and some good views, if there were but a little more method in their arrangement. We extract the following passage from the chapter on Abortion.

"The law of this empire is extremely defective on abortion, for it abounds with the greatest absurdities: its intention is humane and excellent, but it is based upon erroneous physiological principles. It enacts, for instance, that the embryo is not animated until after quickening, that is, until half the period of utero-gestation has elapsed, though the fœtus is alive from the very moment of conception: I have described its developement before the period of quickening, which, I need scarcely observe, could not happen if it were inanimate.

"Again, a jury of matrons is to decide whether a woman be pregnant or has quickened, questions which the whole faculty of physic, in every part of the world, could not determine in the early months of pregnancy: it would be as wise to appoint a jury of infants to determine these questions. The law also enacts it felony to procure abortion before quickening, and subjects the person who does so by any means, or even advises it, to transportation for seven or fourteen years; and to death, if after quickening. Every man must applaud this philanthropic legislation; but it places the medical practitioner in a most dangerous predicament. Thus, in thousands of acute diseases, where life is in the greatest danger, treatment must be employed which may produce abortion; and is the practitioner to allow his patient to die without the benefit which his art affords? In some cases of uterine hemorrhage, the life of the female can only be saved by extraction of the infant; yet this is producing abortion in the eye of the law. Again, if the woman is so far deformed that a full-grown infant cannot be born at the full time, that is, at the termination of the ordinary period of utero-gestation, without a fatal operation, is the medical man to allow the female to be placed in this predicament when he can save her life, and that of her infant, by inducing premature delivery? If the infant arrive at the full term of utero-gestation, it must be destroyed by nature or by art; and by the latter to save the life of the mother: as the statutes now stand, this is felony; but a talented legal writer observes, 'it may be presumed the operator in such cases only commits justifiable homicide, and not the crime of abortion.'" (P. 151.)

We enter our protest against the doctrine inculcated in the following passage regarding *irritative vegetable poisons.*

"This class of poisons seldom comes under the consideration of the medical jurist, and, according to Dr. Beck, 'vegetable poisons are seldom the instruments of murder.' When death is produced by their operation, it generally is caused by suicide or accident, and the coroner's inquest is the only judicial investigation which takes place: besides, the greater portion of these poisons so seldom produces death, that the young jurist need not load his memory with most of them, and he must necessarily be acquainted with the effects of several of them, from his study of the materia medica. A detail of these is evidently unimportant in a compendious work of this description: it is sufficient for our purpose to state, that these poisons have an acrid, sharp, and bitter taste; that they produce nearly the same symptoms as arsenic, mercury, &c.; and that the morbid appearances are nearly the same as those originating from the acrid mineral poisons. The symptoms produced by both classes cannot, in many instances, be distinguished from those arising from diseases." (P. 257.)

It appears to us, that a medical practitioner would be very ill qualified to discharge his duties either to the public or to his patients, if, when called to a case of designed or accidental poisoning, he could only say, "the symptoms arise from some vegetable poison, but really what specific poison I cannot tell, although I am conscious that upon such knowledge ought to depend the nature of the medicines prescribed for relief:" how would such an answer sound from the witness's box? would it redound greatly to the credit or the profit of him who made it? Or, to put a case quite analogous to a case of poisoning by opium, henbane, water-dropwort, mushrooms, fool's parsley, laburnum, hellebore, cherry water, meadow saffron, belladonna, stramonium, foxglove, &c., suppose a person poisoned, designedly or accidentally, by arsenic, corrosive sublimate, or lead, would the patient, or his friends, or a jury be content with the answer that life was endangered, or death induced, by the exhibition of a *mineral poison*, but what specific poison the deponent cannot tell; not because there is any great difficulty in ascertaining which, but because he does not know what ought to be known by all.

There is but one subject more to which we shall allude.

Dr. Ryan states, page 141, that, "in cases of extra-uterine fœtation, should the Cæsarean operation, or rather gastro-hysterotomy, be performed, the infant cannot inherit property according to the laws of this country;" and he gives us as his authority, Blackstone. We wish he had referred to the chapter or section on which he has founded this, we believe, very erroneous doctrine: we know of none that will give it the slightest countenance; for, if the infant live, whether brought into the world "viis naturalibus" or by operation, it will inherit equally; at least, such is our version of the law. Perhaps the author has here confounded direct inheritance with inheritance by *courtesy*, on which, if the mother has real property independent of the father, should the infant be extracted by operation *after* the death of the mother, and die shortly afterwards, the mother's property would go to her relations by blood, and not be possessed by her husband; but should the infant be extracted during the life of the mother, and both subsequently die, the child being no longer then in the previously supposed case, then the husband and father would inherit: if the child lived, as we have before observed, whether brought into this world naturally or otherwise, before or after its mother's death, it would inherit equally. This we believe to be the true exposition of a somewhat complicated point in law.

Upon the whole, we cannot but consider this work of Dr. Ryan's as a feeble and ill-digested compilation. Whether he will accuse us, as he has a contemporary reviewer, of "base personal spite," for thus expressing our honest opinion, we do not stop to consider: we beg to assure him, however, that we earnestly sought to detect enough of redeeming merit in the volume to justify us in passing a more favorable verdict upon it, but our search was in vain. We must remark, that Dr. Ryan does not scruple to adopt the precise language of other writers, without acknowledgment. He, as a reviewer, would complain of such literary pilfering in others, and we cannot permit him to escape without censure. We subjoin one specimen of plagiarism.

Mr. Thomas, in our Journal for November 1828, p. 422, concludes a paper on "Corporeal Organization, &c." in the following words: "In concluding this essay, I beg leave to observe, that the doctrine of the materiality and mortality of the soul should for ever be exploded as totally false, and unworthy of all regard; as subverting the fundamental principles of all religions; as introducing civil anarchy into the political economy of legislation; as substituting discord for harmony, despair for hope, and eternal darkness for everlasting light." At page 16 of Dr. Ryan's work, we have this passage nearly verbatim, with the addition of seven *original* words, which had much better have been omitted, for the reason we have previously stated: "The doctrine of the materiality and mortality of the soul, which is that of materialism and phrenology, should for ever be exploded, as totally false and unworthy of all regard; as subversive of the fundamental principles of all religions; as introducing civil anarchy into the political economy of legislation; as substituting disorder for harmony, despair for hope, and eternal darkness for everlasting light." (*Ryan*, p. 16.) This sentence is given with all the appearance of originality: no inverted commas mark that it was borrowed from another, nor is Mr. Thomas's name mentioned. Dr. Ryan is professedly a compiler, but he is not therefore justified in appropriating to himself the precise language of other writers without acknowledgment.

---

*The History of the Contagious Cholera; with Facts explanatory of its Origin and Laws, and of a Rational Method of Cure.* By JAMES KENNEDY, Member of the Royal College of Surgeons, London.—8vo. pp. 291. Cochrane, London.

*Remarks on the Cholera Morbus: containing a Description of the Disease, its Symptoms, Causes, and Treatment; together with Suggestions as to the best Means of guarding against its Attack; submitted to the Attention of the Medical Profession, but designed principally for the use of the Public in general.* By H. YOUNG, M.D., formerly of the H. E. I. C. Medical Service in Bengal.—8vo. pp. 78. Smith, Elder, and Co., London.

*Practical Remarks on the Disease called Cholera, which now exists on the Continent of Europe.* By JOHN GOSS, Member of the Royal College of Surgeons in London, and late Assistant Surgeon in the H. E. I. Company's Service, Bombay Establishment.—8vo. pp. 27. Longman, London.

*Observations on the Nature and Treatment of the Cholera Morbus, now prevailing Epidemically in St. Petersburg.* By G. W. LEFEVRE, M.D., Member of the Roy. Coll. Physicians, London, and Physician to the British Embassy, St. Petersburg.—8vo. pp. 96. Longman, London.

*Is the Cholera Spasmodica of India a Contagious Disease? The Question considered in a Letter to* Sir HENRY HALFORD, Bart. By W. MACMICHAEL, M.D.—London.

*An actually Practised and effectually Successful Mode of Treatment of the Cholera.* Translated from a Letter of Dr. EWERTZ, Practitioner, of Dünaburg, in European Russia, addressed to Baron E. F. VON GRAEFE, &c.—8vo. pp. 8. Schloss, London.

IF there be wisdom in multiplicity of counsel, or knowledge conferred in proportion to the quantity of writing, there can be no lack of information upon the all-engrossing subject of cholera. We, of course, have felt it our duty to consult all the works which have been written upon the subject, but we caution our readers not to inflict upon themselves such unnecessary labour. Of all the works that have yet appeared upon cholera, Dr. Kennedy's is, in our opinion, the one from which medical practitioners, and the public in general, may derive the most useful and requisite instruction, and from this we shall draw the principal materials for the present article. We must not, however, refuse to other writers (and we would particularly mention Dr. Lefevre and Dr. Hawkins,) the credit of having given us much interesting information respecting this appalling disease. One interesting point, the question of contagion, has also been most ably discussed by Dr. Macmichael: in his letter to Sir Henry Halford, the speculative and fallacious doctrines of the anti-contagionists, not only with respect to cholera, but various other diseases now universally admitted to be contagious, are clearly and satisfactorily refuted.

Dr. Kennedy, during a temporary residence in Bengal, where the cholera originated, had an opportunity of observing the disease to a considerable extent, and of conducting the medical treatment of a number of patients: the results of this experience, and of information obtained in Bengal respecting the cholera, are now submitted to the profession. Our readers will be glad to learn that the curative process of the disease is the particular object of Dr. K.'s attention. Most of the facts contained in his work are confessedly taken from the Reports on Cholera, which were compiled in India by order of the East India Company; and that of Mr. Scot, secretary to the Medical Board of Madras, is referred to, as claiming peculiar respect. Dr. Kennedy gives two maps, which illustrate the geographical progress of cholera, and to render them the clearest and most correct hitherto published, neither time nor attention has been spared.

The variety of Indian cholera under consideration has had several names assigned to it, as the "epidemic cholera," "spasmodic cholera," "epidemic spasmodic cholera," "cholera asphyxia," "malignant cholera," &c. In India, the *species* of cholera to which this variety belongs had existed from the earliest ages, but in 1817 the disease assumed a contagious property, which there is no evidence to prove it ever before possessed, and a name was then wanting to distinguish the new variety. Some writers, convinced of its propriety, have abstained from using the title "contagious cholera," in deference to the opposition of the non-contagionists. Dr. Kennedy, very properly, is not influenced by any such misplaced courtesy: his defence for applying this designation he rests on the facts adduced towards the end of the volume in favor of contagion, and the common and surely rational practice, in physical philosophy, of adopting the theory which best explains the phenomena.

Drs. Barry and Russel have divided the cholera into two stages, the *cold* and *hot* stage. In Dr. Kennedy's arrangement, the *cold* period, or, as he terms it, "acute cholera," is *not* comprised in *one* stage; it is divided into two types, and several stages, for the purpose of medical treatment. To the hot or fever period, and indeed to all the morbid sequelæ of acute cholera, Dr. K. would apply the name of "chronic cholera." The Board of Health, in speaking of the rapid form of acute cholera, observe, "in cases of this severity, the vomiting and purging characteristic of the disease do not commonly take place so early as in milder attacks, but seem to be delayed until the almost overpowered functions of the body make a slight effort at reaction. It is worthy of remark, that, unless death takes place, in these extreme cases, within a few hours, some effort of the animal power is made to rally the constitution; and this point is insisted upon here, because it will direct the mind of practitioners to *the particular moment when bleeding*, and certain other parts of practice, recommended in the Indian Reports, can be enforced in this country with probable success."

To the doctrine here inculcated, Dr. Kennedy objects: judging from the practice in India, he is convinced that it will lead to the loss of numerous lives that might have been preserved under judicious treatment: he says, "here, bloodletting is advised to *wait* upon reaction; and, in the majority of violent cases thus treated, reaction will never occur. No; bloodletting should be performed the instant of the attack, or in as few minutes after as possible; and if blood can be freely procured, the bloodletting will be the efficient remedy in *establishing* reaction. It may appear improper thus early in our analysis to refer to any point concerning the treatment of the disease, but we were unwilling to pass over the above remarks, which Dr. K. makes in the preface to his work.

In the first section of the volume, Dr. Kennedy gives a well-written description of the early impressions which are created on entering Bengal; the peculiarities of the climate; the origin of the malignant variety of cholera in, 1817, which is ascribed to the climatic vicissitudes and peculiarities of Bengal; the propagation of the disease through the upper provinces of Hindoostan by contagion, and the progress of the contagious cholera during the first twelve months of its course. In the southern parts of Bengal, the prevailing winds are north and south. The northerly and southerly winds blow alternately during unequal portions of the year, over that quarter of the province which approaches the ocean. The seasons conform nearly with those changes of the prevailing winds, and are usually distinguished by the terms hot, rainy, and cold. The hot season commences with the approach of March, the thermometer ranging between 73° and 86°, and the heat gradually increases to the latter end of May, when the weather has become exceedingly sultry and oppressive. The thermometer now fluctuates between 81° and 93° in the shade, and the nights are calm, close, and suffocating. The rainy season sets in about the middle of June, and with it, to the relief of the parched inhabitants, the atmospheric temperature declines. The cold season commences in November and ends in February: "it is the period of enjoyment in India." When the seasons observe this general course and period of succession, their influence on the constitution of the inhabitants is productive of several maladies, which are considered in a great measure peculiar to those different divisions of the year. Rheumatism, catarrh, ague, and diarrhœa, attend the cold season; the scorching heats of summer are productive of bilious remittent fever, which continues until August or September, when bilious dysentery becomes the prevailing distemper. Cholera, and acute inflammation of the liver, may occur in any month of the year, but they appear most frequent about the beginning of the rains.

"In irregular seasons, and such will occasionally occur, the rains set in much earlier or later than is usual, forming what is called a dry or a wet year. Under these circumstances, the climatic affections are increased both in frequency and severity, and their characters will be determined by the nature of the atmospheric vicissitude. If the hot summer weather have been protracted, the enervated population suffer in an aggravated degree from the distempers common to hot seasons. If moisture prevail, the maladies of the rainy season may be anticipated in a more than ordinary abundance.

"These are the evil effects succeeding directly to the influence of an irregular season; but there are others of still greater magnitude, which spring indirectly from the same source. Irregular seasons, for instance, deteriorate the produce of the earth, and consequently the health of the inhabitants will be deteriorated by the use of the diseased grain, and the scarcity of food which necessarily follows a bad harvest. An extreme example of this was witnessed in the dreadful famine and its attendants which, about the year 1769, carried off three millions and upwards of the people of Bengal. The falls of rain had been unfrequent and of short duration, so that every plant was parched and unproductive. The grain crop was almost a total failure; and as the two former crops had been scanty, in their hour of extremity, the inhabitants had no resource. Rice soon attained ten times its usual price, and the miserable people were driven by the cravings of hunger to the woods, where they perished in thousands, after devouring the bark of trees and the remains of putrefying vegetables.

"It is during the existence of these extraordinary calamities that mankind are most exposed to the inroads of pestilence. The scarcity and badness of the food, together with the deranged state of the atmosphere, conspire to debilitate and corrupt the animal system. Whole families, in a state of utter destitution, ultimately become, through bodily weakness, unable to leave their beds or hovels, and there they lie surrounded by accumulating filth, until hunger or disease puts a period to their sufferings. It is not surprising to find, therefore, that many maladies previously known only in a mild shape, should, in such a concentration of misery, become exceedingly virulent, or that some non-contagious distemper, occurring casually at the time, should suddenly assume a contagious form." (P. 11.)

The close alliance existing between bad food, atmospheric vicissitudes, and the growth of contagious diseases, says Dr. K., has been demonstrated in Bengal, even of late years. During the rainy season of 1815, the fall of rain was excessive; the hot season of 1816 was distinguished for drought and intense heat; the year 1817 was marked with as singular deviations from the ordinary course of the seasons as those of the preceding year. Between these two annual periods, a further resemblance is afforded in the fact, that while 1816 gave birth to the contagious disease called "malignant sore throat," in 1817 originated the contagious cholera. Dr. Kennedy points out the importance of bearing in mind that a milder and non-contagious form of cholera had been known for centuries in Bengal; and he states, that the occurrence of these non-contagious cases in 1817 and succeeding years was often confounded with that of the malignant kind, and hence many contradictory but still reconcileable statements.

"It appears that during the month of August, 1817, a pretty severe type of cholera broke out among the natives of Bengal, at or about the same time in different parts of the country, and in places where there had been no inter-communication. This shews to conviction that the state of the atmosphere, and the bodily condition of the native population in general, were circumstances sufficient of themselves to change the character of cholera; and that the virulent variety of the disease is not to be referred to any known or unknown cause, operating only in a particular locality of the province.

"But when a malady originates in the way just described, its intensity is greatly modified by local circumstances. In a village supplied with running water, and surrounded by open grounds, which are favorable to free ventilation, the type will be less dangerous than in a town crowded with inhabitants, hemmed in by jungle, and exposed to the exhalations of stagnant water. The type prevalent in the village, indeed, may be decidedly non-contagious; while that in the town (the disease being the same in kind, but different in degree,) may assume the most virulent and contagious form." (P. 19.)

Of the latter form, and distinguished by its intense malignity from all other varieties, was the Indian cholera which commenced in the town of Jessore, in August 1817. "Jessore is a crowded, dirty, ill-ventilated place, surrounded by a thick jungle, and exposed during the rains to the effluvia of an immense quantity of stagnant water." In the short space of a few weeks, 10,000 of the inhabitants perished in the single district of Jessore. We regret that we cannot follow Dr. Kennedy through his admirable description of the rise and progress of the disease in other directions.

The second section of the work consists of very interesting abstracts taken from the Medical Reports published in India by order of the government. In these documents, concise descriptions of the cholera are given, and the various opinions of different writers are condensed. From an attentive perusal of these abstracts, we perfectly agree with Dr. Kennedy, that the reader of them "cannot fail to have observed a few facts of paramount interest, which, by the concurrent testimony of a variety of witnesses, far apart, and ignorant, at the time, of each other's views, seem to have attained the rank of general facts.

"These general facts, in characterising the contagious cholera, shew that it is extremely fatal and rapid in its course; that it may exist in various degrees of severity in different individuals, and in various degrees of severity in different localities, and at different times in the same locality; and that, in the treatment pursued, bloodletting, calomel and laudanum, brandy, arrack, and other spirits, and the application of moist or dry heat to the surface of the body, were the grand remedial agents." (P. 159.)

Dr. Kennedy particularly dwells upon one source of great confusion and contradictory testimony respecting the treatment of the disease. Many remedies, on which the strongest dependence is to be placed in the management of cholera, have repeatedly fallen into temporary disgrace, from their having been prescribed in stages of the disease when their use was altogether improper. For the purpose of medical treatment, Dr. K. divides the disease into two types, or varieties, the *protracted* or severe type, and the *rapid* or violent type.

"*Discriminative Treatment of Acute Cholera. Protracted type.* The protracted type consists of two stages; and, in order that the practitioner may easily recognize these stages respectively when he meets them, we shall enumerate a few of their symptoms beyond those of practical value.

"*First stage.* The patient complains of feeling of anxiety, or of uneasiness at the pit of the stomach. After some time, nausea supervenes, and the uneasiness changes into a feeling of heat or pain. To these symptoms succeed vomiting and purging, and prostration of strength. The evacuations, at first, consist of the common contents of the alimentary canal; afterwards of a fluid like ricewater. Occasional cramps are felt in the limbs; the pulse is small and rather quick; the skin feels a little cold, and the temperature is gradually decreasing; the countenance of the patient is somewhat shrunk, and the features appear sharper than natural.

"If the disease be left to itself, or if it continue to advance in spite of the remedies that may have been used, the symptoms increase in severity, and the patient comes to suffer from violent cramps in the upper and lower limbs, and at times in the muscles of the chest and belly: the cramps, in general, are not constant; they recur at short intervals, in paroxysms. The vomiting and purging are severe. The coldness of the skin has increased much:

it feels moist, and is of a bluish colour about the face, hands, and feet; the palms and soles of the latter appear corrugated, as if they had been steeped in water. The pulse is barely, or not at all, to be detected in the wrists and temples; the countenance is ghastly, and expressive of great anxiety; there is distressing thirst, and burning heat or pain in the region of the stomach or bowels.

"If the disease be still uncontrolled, it will pass into the second stage.

"*Second stage.* Under the increasing debility, the vomiting, purging, and cramps are subsiding or have disappeared. The patient lies in a state of helpless exhaustion, and is almost incapable of making the slightest movement. He is apparently insensible, but as his senses remain unimpaired to the last, he may be roused to say 'Yes' or 'no.' The pulse is gone, and even the action of the heart is extremely feeble; the surface of the body is deadly cold; the breathing is oppressed or scarcely perceptible, and the countenance is quite cadaverous." (P. 173.)

In the treatment of the first stage, the first object is to allay the cramps, vomiting, and purging. No single remedy should be relied upon. The warm bath, bloodletting, and a large dose of calomel and laudanum, should be given immediately. The remedy first at hand should be administered first. As soon as the cramps are subdued or checked, the patient should, with all possible expedition, be removed from the bath, and placed between *dry* heated blankets. Dry warmth should be further afforded by surrounding the body and limbs with bags of heated sand. If the cramps do not return, new measures will be required, to remove debility, and to excite the natural action of the bowels. In the second stage, we have two principal indications: 1st, to remove the deadly coldness of the body; 2d, to remove the great debility. The coldness must be attacked by the application of *dry* heat: for the removal of the excessive debility, ether, brandy, or other spirits, hartshorn, &c. are to be had recourse to. If reaction takes place, then these remedies may be dispensed with.

"*Rapid type.* In many instances the attack of cholera commences with very aggravated symptoms: the patient falls suddenly down in a state of extreme weakness, and perhaps temporary insensibility, and the energies of the constitution are so much oppressed by the influence of the morbific poison, that there may be little or no visible attempt at reaction. The pulse almost instantly, or in an exceedingly short period, disappears at the wrist, and the heat of the body rapidly declines. In these cases, cramps, vomiting, and purging are either completely absent, or an attempt to establish these symptoms is only evinced in slight spasms, and the occurrence of one or two nearly passive evacuations. The violence of the disease, however, in this type, and the uniform course which the symptoms are inclined to pursue until death closes the scene, should not deter us from the application of a discriminative method of medical treatment. The acute symptoms of the rapid type may be divided into two stages: one, of apparent debility; the other, of real debility.

"The chief remedial measure is bloodletting, and here its use is founded upon the fact, that, for a certain space of time after the commencement of the attack, the resources of the constitution are not to be measured by the external symptoms. Apparently the patient is in a state of hopeless debility, (and this will generally prove to be the case if he is left to the efforts of nature alone,) but when properly assisted by art, the constitution is often enabled to rally, and eventually to recover from the shock. The time, however, in which this medical service may be performed with the greatest hope of success, is unfortunately extremely limited; in some cases not exceeding many minutes after the patient has been seized.

"If the practitioner arrive before the blood has deserted the superficial vessels, bloodletting should be performed to the extent of twenty or thirty ounces in an adult; then other auxiliary measures will be required: of these, nothing should supersede the immediate and continued application of dry heat to the surface of the body. The value of this remedy is incalculable, and it has the advantage of being applicable at any period; whereas the opportunity for the abstraction of blood may be soon irrecoverably lost. Internal stimulants, as ether, hartshorn, &c. should be also administered, in order to expedite the establishment of reaction. If the second stage, that of real debility, have commenced before the patient is seen, the treatment will consist of dry heat and internal stimulants, without bloodletting." (P. 178.)

In the abstracts of the Indian Reports, instances may be observed in which the warm bath, used in the second stage of acute cholera, seemed to hasten the death of the patients; and, in Dr. K.'s opinion, no form of *moist* heat should ever be used in acute cholera, unless at the commencement of, or during the prevalence of severe spasmodic symptoms. "That moist heat should have a dangerous influence on patients in a state of *real* debility, appears very natural, when it is recollected that even persons in health will faint after long immersion in a bath at a high temperature." Large doses of laudanum or calomel are said to be useful only during the symptoms of the first stage of the protracted type. Bloodletting is to be avoided towards the termination of the first stage of the protracted type, during the whole of the second stage, and in the rapid type during the period of real debility. Dr. Kennedy gives many other important directions respecting the treatment of cholera, for which we must refer to the work.

The progress of the cholera exterior to Hindoostan is next traced.

"In Russia the cholera observed the same laws that had marked its progress in India and other countries. Adhering for some time to the route of navigable rivers and high roads, it attacked, in the first instance, the boatmen, the travellers, and the towns situated on either side of these lines of general communication." (P. 213.)

The *Laws of Cholera* are comprehended by Dr. K. under the following designations.

"1st. *Climactic influence.* The contagion of cholera may spread in every climate, with its spreading powers but slightly, or not at all, impaired.

"2d. *Predisposition.* Persons in certain states of bodily health are peculiarly liable to be attacked.

"3d. *Latent infection.* The period of time during which the contagion lies dormant in the system rarely exceeds three days.

"4th. *Increase and decline.* When the cholera appears in a town, it extends rapidly, and, in general, runs through its course in the space of a few weeks.

"5th. *Contagion.* Cholera is contagious, and its contagion is of a highly diffusible nature." (P. 215.)

That the disease is really influenced and modified by the existence of these laws, Dr. Kennedy very clearly proves; and upon the subject of contagion, his evidence is equally satisfactory. He completely refutes the chief arguments of the anti-contagionists, and shews also, by indisputable facts, that a contagious property must be assigned to the disease.*

Under the head of *Quarantine*, Dr. K. points out the proper precautions to be adopted to prevent the introduction and spread of cholera in this country.

The Appendix to the work contains a general summary of the symptoms of cholera, as given by Mr. Scot, the author of the Report of the Medical Board of Madras.

Dr. Young is a non-contagionist: the principal argument he adduces in support of his belief is, that many have been exposed to cholera, and have not been attacked. This often-repeated argument cannot now require any formal reply: the same mode of reasoning would shew that smallpox is not contagious. Mr. Goss also "acknowledges himself a disbeliever" in the contagious powers of cholera, but he is by far the most prudent anti-contagionist we have met with: he wisely admits that, "while there exists the possibility of doubt on the subject, it is incumbent that every precautionary measure be used to avert the invasion into this country of so dreadful a calamity."

We received Mr. Orton's work on the Indian Cholera too late for notice this month, but we shall give an analysis of it in our next number.

* In our Intelligence department, we have adduced additional evidence, from various authorities, in proof of the contagious nature of cholera.—Ed.

*A Treatise on the Diseases of the Heart and Great Vessels, comprising a New View of the Physiology of the Heart's Action, according to which the Physical Signs are explained.* By J. Hope, M.D., Member of the Royal College of Physicians, &c.—8vo. pp. 600; seven diagrams. W. Kidd, London.

Morbid anatomy has now so well established its claim to rank high in the essential studies of the medical practitioner, that there is little cause to fear that it will ever again fall into neglect. The brilliant discoveries of the continental schools, confirmed and extended by a few zealous investigators at home, have, in the eyes of all candid judges, given such a balance in its favor that, in essays or lectures on a disease, the post-mortem appearances now require as conspicuous a place as the *methodus medendi*, or indications of cure. We observe with pleasure the anxiety with which the students press to the anatomical table of our metropolitan hospitals, accompanied by the candid and philosophic physician, who disdains not to join them in taking lessons still from nature. We hail as a favorable omen of the extension of this spirit, the numerous reports from hospitals and private practice in the country with which our Journals abound, and which exhibit a minuteness of research and a familiarity with principles which, half a century ago, would scarcely have been intelligible. How different, in fact, was the rare and uninteresting operation of "opening a body" in those days, from the careful scrutiny of organ and tissue by which the physician now studies the nature of those agencies which it is the object of his art to counteract!

Nevertheless, there are evils in an excessive devotion to this study; and, although we cannot join in the impatient (we had almost said, mercenary,) cry of those who call for practice and a recipe from every step in science, yet we would warn the student lest, fascinated by a pursuit in which he finds himself on a par with his seniors, and may not unfrequently be led to detect their errors, he neglect his part as an actor for that of a critic, and have the dissecting room instead of the sick chamber for his proper sphere of action. Here, as we conceive, is the shoal on which a large number of the students of the French schools become stranded, when launched into practice. Highly instructed in the ultimate effects of disease on tissues, yet imperfectly acquainted with the previous train of symptoms, biassed by the nomenclature of their schools against all things that they call irritants in diseases which their anatomical examinations have decided as inflammatory; yet utterly unacquainted, *practically*, with the remedial agents which they condemn, their practice is either timid and inefficient, or partial and incomprehensive of the multifarious functions and powers of the complex frame. Even the disciples of the doctrine of physiological medicine, professing (as they do) to follow nature for their guide, are so warped in their ideas by a language far too generalizing and dogmatic for a comprehensive consideration of disease; and therefore become better orators in a class-room than physicians at the bedside. Thus the French school of medicine, although better founded, and possessing a more ample field for scientific instruction, than our own, turns out men much less qualified for practice.

We have said that the study of morbid anatomy is seducing, and, in a spirit of apprehension of the evils of its abuse, we commenced the reading of the work before us, when we found that, after the manner of Laennec and other French writers, the author begins his description of diseases by an exposition of their anatomical characters. In a general way, we do not consider this a desirable method for a work of practical medicine: however advantageous it may have been for Laennec, and other original cultivators of the field of pathological anatomy, to bring forward most conspicuously that part which most distinguishes their works, we should be sorry to see this inverted arrangement supersede that which follows the course as well as the exactitude of nature, and which, by introducing the student to the clinical history of the disease, afterwards illustrated and confirmed by the anatomist's scalpel, accustoms his mind to a closer study of that *living* pathology, which is, in fact, the important object of his contemplation.

But we were pleased to find, on further perusal of Dr. Hope's work, that ardor for morbid anatomy had not withheld him from the bedside; and, in the ample and interesting details of the general and physical signs, exciting causes, and treatment, we were glad to hail the labours of a physician for the living, as well as a scientific registrar of the dead. We consider, therefore, that we shall not enter on an unprofitable task in devoting a few pages to review a work which professes to treat, in a comprehensive and practical manner, of the diseases of what may be termed the most essentially vital of all the organs of the body.

Dr. Hope is already known to the public by his experimental researches on the physiology of the heart's action, in which he endeavoured (and certainly with better chance of success than in hypothetical disputes,) to settle the contested question of the causes of the sounds of the heart's pulsation. The unsatisfactory state in which Laennec left this and several other points relating to the heart and its diseases, the want of any other recent work on the subject in the English language, and the partial and over-generalizing character of that of Bertin and Bouillaud, leave a space for further research and experience, and almost render unnecessary the apologies with which Dr. Hope modestly presents his treatise to the public.

An introductory part prefixed to the volume exposes pretty fully the objects of the work, and, in a summary of the defective points in preceding writers, and of the peculiarities of his own views, the author sets forward his claim to attention. The following paragraph, if verified by the context, will be allowed, by all who know the difficulty of distinguishing, and therefore of successfully treating diseases of the heart, to shew an important step in this department of practical medicine.

"Many think that the expectation of effecting an improvement in diseases of the heart chimerical; and this, because, not being accustomed to recognize them before they have obtained an advanced stage, they are preoccupied with the old and popular idea of their incurability. To such it might, perhaps, be a sufficiently philosophical answer to reply, that an improved knowledge of the nature and causes of a disease must, alone, necessarily lead to improvement in the treatment, and that therapeutic weapons are dangerous when wielded in the dark. But here we may go much further: we may say that, by the improved means of diagnosis, the maladies under consideration may be recognized, not only in their advanced, but in their incipient stages; and even when so slight as to constitute little more than a tendency. We may say, on the grounds of incontestible experience, that, in their early stages, they are, in a large proportion of instances, susceptible of a perfect cure; and that, when not, they may, in general, be so far counteracted, as not materially, and sometimes not at all, to curtail the existence of the patient. We may accordingly predict that the term 'disease of the heart,' which at present sounds like a death-knell when whispered by the physician, will hereafter become, by familiarity, not more alarming than the term asthma, under which it is frequently disguised." (*Introduction.*)

The author further urges the immediately practical importance of his subject, in the cases of tendency to apoplexy from disease of the heart, of palpitation attributed to the stomach, &c. Here, in ignorance of the real cause, active exercise is enjoined, and often with fatal results. Again, in the converse of these cases, in those, namely, in which disordered digestion or hysteria simulates diseases of the heart, patients are often alarmed and subjected to a painful course of treatment, through ignorance of a distinction which our author says may be made 'with ease and certainty.' After this our readers will excuse further apology, and will accompany us in our glance at the work with as much attention as they can spare from *cholera.*

The work is divided into six parts: 1, Anatomy and Physiology; 2, Inflammatory Affection; 3, Organic Affections; 4, Nervous Affections; 5, Miscellaneous Affections; 6, Cases.

The chapters on the *Anatomy and Physiology of the Heart* are in many respects interesting and original, and, as they are essential for the explanation of the signs and history of the diseases which follow, they properly become a part of a practical work. The following description of the precise position of the heart in relation to the surrounding parts, will be highly useful in directing the student in his investigation of the physical signs.

"As the apex and body of the heart are free, while the base, secured by the great vessels, is comparatively, though not absolutely fixed, the organ turns in a slight degree upon its base with each alternate movement of the diaphragm, the descent of the muscle causing its longitudinal axis to assume a more vertical position, and the ascent throwing it transversely to the left. It is necessary, therefore, that the auscultator fix upon some given point at the base, which may serve as a mark and guide for his exploration of the situation of the organ. The point which to myself has appeared the most certain, is, the pulmonary artery. This vessel, midway between its origin and the place where it divaricates into the two trunks distributed to the lungs, bulges at the interspace between the second and third left ribs close to the sternum, a circumstance which, as well as the situation of the other parts of the heart, I have carefully ascertained by forcing needles through the thoracic walls, at given points, into the viscera beneath. The situation of the pulmonary artery was, also, well displayed by the dilatation of that vessel described in Case xx. (Weatherly.) A line drawn from the inferior margins of the third ribs across the sternum, passes over the pulmonic valves a little to the left of the mesial line, and those of the aorta are almost directly behind them. From this point the aorta and pulmonary artery ascend; the former inclining slightly to the right, coming in contact with the sternum when it emerges from beneath the pulmonary artery, and following, or perhaps rather exceeding, the mesial line, till it forms its arch; the latter, which is, from the first, in contact with the sternum, inclining more considerably to the left, until it arrives at the interspace between the second and third ribs above described. A vertical line coinciding with the left margin of the sternum, has about one third of the heart, consisting of the upper portion of the right ventricle on its right: and two thirds composed of the lower portion of the right ventricle, and the whole of the left, on its left. The apex beats between the cartilages of the fifth and sixth left ribs, at a point about two inches below the nipple and one inch on its sternal side.

"The lungs descend along the margins of the sternum about two inches apart, and overlap the base of the heart, slightly on the right side, and more extensively on the left: then, receding from each other, they leave a considerable portion of the right ventricle, and a less extent of the lower part of the left, in immediate contact with the sternum.

"The right auricle is in front of the heart, at its right side and upper part. One portion of it is overlapped by the right lung, and another, principally the appendix, is in contact with the sternum. The left auricle is situated deep behind and to the left of the heart at its upper part, opposite to the interval between the cartilages of the third and fourth ribs. The extremity of the appendix is visible in front, but, when the volume of the heart is natural, it is not in contact with the sternum, being considerably overlapped by the left lung. The pericardium ascends on the great vessels as high as the commencement of the arch of the aorta, and opposite to the second ribs.

"When the heart is enlarged, its longitudinal axis becomes placed more transversely, and its lateral diameter is increased. Hence, the right ventricle projects more considerably to the right, sometimes under the whole breadth of the sternum; and the left extends far beyond its usual limits to the left, sometimes elevating by compression that portion of the lung which overlaps it, so as to bring nearly its whole surface, and the tip of the auricular appendix, in contact with the sternum. In addition to being broader and placed more transversely, the organ descends lower than natural, its apex sometimes beating between the sixth and seventh ribs, and its pulsation extending to the epigastrium.

"When the right auricle is dilated or gorged, it extends upwards and to the right, and comes more extensively in contact with the sternum.

"When the pericardium is distended to the utmost with fluid, it forms a pear-shaped bag, the top or narrow extremity of which sometimes mounts even above the second rib: its sides are nearly in contact with the sides of the heart, while its front is separated from the anterior surface of the heart, in the dead subject, by two or three inches of interposed fluid." (P. 3.)

Then follow some directions for the application of percussion, which Dr. H. recommends to be made mediately, either on the back of the fingers, or on the *plessimeter* of M. Piorry. This method of investigation is assuredly of great value in measuring the volume and position of the heart.

Laennec, it is well known, attributed the first sound heard in the heart's pulsation to the impulse of the ventricles, and the second sound to the auricles. The fact noticed by Haller and others, that the auricular *preceded* the ventricular contraction, led Mr. Turner to call in question the latter part of this opinion; and although many theories were proposed to explain the second sound, it remained a matter of doubt when our author commenced his experimental investigation. An account of these experiments, which were witnessed by several distinguished physicians and surgeons, is introduced: they are chiefly those which have already appeared in the Medical Gazette. The conclusions to which they led form the grounds of the exposition of the heart's action contained in the next section. The first motion of the heart is the auricular systole, which is slight and brief, unaccompanied by sound, and transmitted in a somewhat vermicular but rapid motion to the ventricle, which thereupon contracts suddenly, synchronously with the first sound and the impulse against the ribs. The arterial pulse is also synchronous with it, except in vessels at a considerable distance from the heart, as the radial, in which it follows at a barely appreciable interval. The systole of the ventricles is immediately followed by their diastole, which is accompanied by the second sound and an influx of blood from the auricles.

"The rhythm of the heart, that is, the duration of the several parts of this series, which constitute what may be called a beat, is the same as described by Laennec: viz. 1. The ventricular systole occupies half the time, or thereabouts, of a whole beat. 2. The ventricular diastole occupies a fourth, or at most a third. 3. The interval of ventricular repose occupies a fourth, or rather less, during the latter half of which the auricular systole takes place." (P. 41.)

The author offers some interesting remarks on the causes, mechanism, and objects of the motions, and explains some doubtful points which had been hitherto left undetermined by physiologists. The sounds heard during the systole and diastole of the ventricles, Dr. H. ascribes to the motions of the contained fluid.

"All the phenomena of the heart's action, both in health and disease, lead me to believe that the sounds are occasioned by the motions of the contained fluid, and the mechanism of their production I conceive to be, according to the laws of physics, as follows. When the ventricles contract, an impulse is given to the particles of fluid in contact with them; and this being propagated by collision from particle to particle, generates sound." (P. 48.)

"The auricles do not contribute to the production of either of the sounds; as, in the experiments alluded to on the ass, they were heard in equal perfection when the auricles were in a state of immobility." (P. 49.)

We do not think that Dr. Hope has completely established these points, as far as relates to the physical causes of the sound. There is a sound produced in all other cases of rapid muscular contraction, independently of any fluid; and whatever may be its physical cause, we do not see why it should not equally accompany the brisk, vigorous systole of the heart. The impulse of the contained fluid may be a concurrent cause: but inasmuch as motions of this fluid, in certain morbid states, are less questionably known to produce other sounds, (bellows and sawing murmurs,) without changing the natural sounds of the heart, it is more reasonable to suppose that this has a different origin. Dr. Hope rejects this opinion, on the grounds that the natural sounds of the heart are different in character, and louder than the loudest muscular sounds that can be produced by any exertion of the most powerful muscles in the body: but we would remark, that the muscular sounds are not, any more than those of the heart, in proportion to the strength or size of the muscle, but rather to the briskness and extent of its contraction: hence, rapid motions of the fingers, or of the panniculus carnosus in quadrupeds, produce a louder sound than the slower contraction of a much larger muscle; and the heart, in this view, presents the same phenomena in case of disease, the sudden systole of a dilated ventricle exceeding in its sound the labouring and prolonged contraction of one affected by hypertrophy. This matter is, therefore, still open to discussion: fortunately, however, it is not a point of much practical importance, as it does not extend any obscurity to the signs of disease.

The pathological phenomena of the heart's action are next generally described; and here, as may be supposed, the author's views cause him to differ in some respects from the opinions of the discoverer of auscultation: these differences are not, however, so great as might be expected, having reference chiefly to the exclusion of the auricles as causes of sound.

On the subject of *valvular disease*, Dr. H. has some interesting additions to the signs described by Laennec.

"When the aortic or the pulmonic valves are contracted, a morbid murmur accompanies the sound of the ventricular systole; and when the valves, not closing accurately, admit of regurgitation, a murmur accompanies the sound of the ventricular diastole also; but in the latter case it is extremely slight and brief, because, as I imagine, the swift influx of blood from the auricle during the diastole, almost instantly puts an end to any regurgitation capable of producing sound. When the mitral or the tricuspid valve is contracted, a murmur accompanies, and sometimes entirely supersedes, the second sound, being occasioned by the obstructed passage of the blood from the auricle into the ventricle during the diastole of the latter. When the valve, not closing accurately, admits of regurgitation, a murmur accompanies the first sound. This fact was one of the very few overlooked by that wonderfully accurate observer, Laennec. It was noticed by the writer in 1825, and the number of cases with which he is enabled to substantiate it, leads him to assume it as certain." (P. 5.)

"Valvular murmurs are occasioned by collision of the particles of the blood, when this fluid is, by any cause, thrown into preternatural commotion during its passage through the orifice of a cavity." This opinion, which had already been advanced by Dr. Williams,* Dr. H. considers as proved by analogy, as well as by the circumstances of the cases in which these murmurs have been found to occur.

"To offer an experimental exemplification of this, a similar murmur is produced when water is transmitted with sufficient velocity through a tube, in any part of which there exists an internal prominence or contraction of its caliber. The same occurs when the leathern pipe of a fire-engine is slightly compressed with a finger; or, when similar compression is exercised with the stethoscope on a superficial artery of primary or secondary magnitude.

"Obstructions of a hard, rugged kind, as ossifications, occasion louder murmurs than smooth obstructions, because they more completely break the current of the blood. Murmurs are not, as is often supposed, louder, *cæteris paribus*, in proportion as the valvular contraction is greater. On the contrary, the loudest murmurs are produced by a moderate contraction, and they become weak when it is extreme. Thus, a rugged osseous concretion, the

* Rational Exposition of the Physical Signs of Diseases of the Lungs and Pleura; 1828. P. 50.

size of an ordinary pea, in the aortic orifice, I have found to produce the loudest possible murmur; whereas, a contraction of the mitral or tricuspid valve to the size of only two, three, or four lines in diameter, I have frequently known to occasion little or no murmur. Osseous asperity, alone, without contraction, produces considerable murmur. From the above premises, it may be stated as a general principle, that the loudness of a murmur is in proportion, not only to the roughness of the obstacle, but also to the quantity of fluid transmitted through the valve, and put in preternatural commotion by the obstacle. The effect we should naturally expect to be aided by the force and velocity with which the fluid is impelled; and, accordingly, we find that when the ventricle is hypertrophous, or the circulation hurried, the murmur is proportionably louder." (P. 56.)

Dr. H. conceives that murmurs may also depend, in some measure, on the configuration and position of the orifices, and, in illustration, introduces some hydraulic diagrams from Venturi, to shew the manner in which various currents and eddies are produced in fluids discharged from vessels and apertures of a particular formation. By these illustrations he explains the production of valvular murmurs by a funnel-shaped auriculo-ventricular orifice, by a tense valve with a central perforation, by regurgitation through a projecting valve, &c. In a case which presented itself to Dr. H. in 1825, he was led to notice that a murmur was produced by a disproportion between the cavities and orifices, consequent on enlargement of the former. This phenomenon, of great value in diagnosis, he likewise ascribes to altered direction of the currents of blood, which become sonorous in their preternatural motions. For the detail of these opinions we must refer to the work, with the remark that they are not matters of merely speculative curiosity; for an accurate knowledge of these physical signs will give us a clue to the diagnosis of many diseases of the heart, still matters of acknowledged difficulty.

It appears, however, that even these sounds or murmurs may be produced in the heart and arteries, independently of organic disease, and our author has taken considerable pains to investigate the circumstances under which they occur: he points out several inconsistencies in the opinions of Laennec on this point, which to us have always appeared unsatisfactory. In fact, those who had intercourse with M. Laennec during the last year of his attendance at La Charité, must have perceived that his own mind was not clear as to the causes of the phenomena in question. He attributed the bellows murmur to the muscular sound occasioned by a spasmodic contraction of the muscular fibres of the arteries: now, it is quite certain, if any such spasm ever take place, that it cannot be of an extent or kind equivalent to produce the phenomena in question. We have before remarked that muscular sound is generally proportioned to the briskness and extent of the contraction: now, the bellows sound heard in the arteries is often louder than even the sound of the heart itself, or than any known example of muscular sound. With respect to the purring tremor and thrilling pulse, Laennec avowed his uncertainty. On these points, Dr. Hope advances as follows:

"As my own experience does not accord with that of Laennec as to which motions of the heart are accompanied by the murmur, it is necessary to premise that I have found it accompany the systole of the ventricles, and not the diastole, unless it, at the same time, and in a predominant degree, attended the systole. In the arteries, it coincides with their diastole. The purring tremor occurs at the same moment, and is a result of the same cause. The arterial thrill is nothing more than a less degree of the purring tremor.

"Both by experimental and pathological evidence, I am led to believe that the murmurs and tremors, as well in the heart as in the arteries, are occasioned by modifications in the motion of the fluid." (P. 69.)

He then proceeds to prove that liquids permeating tubes with a certain velocity, do produce sounds according to ascertained physical laws; and then cites some experiments, performed in conjunction with Dr. M. Hall on dogs, to prove that certain modifications of the circulation, succeeding to loss of blood in this way, occasion the various phenomena of purring tremor, bellows and sawing sounds, and jerking pulse, the impulse of the heart being unusually smart and abrupt. These phenomena, in these examples connected with the reaction from loss of blood, the author considers to arise in other cases from the same principle. Thus, in those affected with pericarditis and adhesion of the pericardium, and in young people of plethoric habit and delicate irritable temperament, subject to nervous palpitation, the bellows sound, or sawing murmur, is caused by a peculiarly jerking or convulsive action of the heart, which throws the current of the blood into unusual sonorous vibrations.

"In the latter class the murmur may come on whenever the circulation is excited, and, for exciting it, the most trivial causes are sufficient. I have seen a single cough, or a full inspiration, or a little flatulence, produce the effect for a few beats only; while the act of turning in bed, of rising suddenly, of being startled by any noise, has occasioned it for several minutes. An emotion of grief or pleasure will sometimes produce a more considerable and permanent effect. I have often been assured by patients that the momentary flash of an idea across the mind, has sufficed instantly to excite a violent fit of palpitation, and that this has recurred several times a day, whenever the same idea has presented itself. That, under so irritable a state of the nervous system, the heart should contract with spasmodic abruptness, might be anticipated. What theory points out, experience proves; for the sharp, jerking pulse and beat of the heart of a patient in a state of nervous agitation, is too well known to require demonstration. Sometimes, when the nervous excitement is excessive, a violent throbbing is perceptible over the whole body, and the bellows murmur and thrill are distinct in every considerable arterial trunk. When such is the case, the anxiety and distress of the patient are extreme, and his situation is not exempt from danger. Though death is rare when the symptoms are purely nervous, I have once seen it occur. The patient, a healthy plethoric young female, who became affected on the intelligence that her husband had deserted her, lay twenty-four hours in a state of almost complete insensibility, and the violently bounding, jerking and thrilling arterial throb, together with universal flushing, heat and perspiration of the surface, resisted every remedy, and only subsided with the wane of life." (P. 76.)

(To be concluded in our next.) volume, p. 153.

